# Fibrinogen is an independent preoperative predictor of hospital length of stay among patients undergoing coronary artery bypass grafting

**DOI:** 10.1186/s13019-023-02238-w

**Published:** 2023-04-07

**Authors:** Chunsheng Huang, Wenyuan Zhang, Xiaofei Chen, Xia Xu, Jun Qiu, Zhihao Pan

**Affiliations:** 1 Department of Anesthesiology, Ningbo Medical Center Lihuili Hospital, Medical School of Ningbo University, Zhejiang, 315040 China; 2 Department of Anesthesiology and Intensive Care, The First Affiliated Hospital, School of Medicine, Zhejiang University, Hangzhou, 310003 China

**Keywords:** Fibrinogen, Propensity score matching, Hospital length of stay, Coronary artery bypass grafting, Postoperative complications

## Abstract

**Objective:**

This study aims to examine the impact of preoperative fibrinogen concentration on the short-term outcomes and hospital length of stay (LOS) of patients undergoing Coronary Artery Bypass Grafting (CABG).

**Methods:**

Between January 2010 and June 2022, a retrospective analysis comprised 633 patients who sequentially received isolated, primary CABG. These patients were categorized into normal fibrinogen group (fibrinogen < 3.5 g/L) and high fibrinogen group (fibrinogen ≥ 3.5 g/L) according to preoperative fibrinogen concentration. The primary outcome was LOS. To correct for confounding and investigate the effect of preoperative fibrinogen concentration on the short-term outcomes and LOS, we employed propensity score matching (PSM). The correlation between fibriongen concentration and LOS in subgroups was examined using subgroup analysis.

**Results:**

We categorized 344 and 289 patients in the “normal fibrinogen group” and “high fibrinogen group”, respectively. After PSM, compared to the normal fibrinogen group, the high fibrinogen group had a longer LOS [12.00 (9.00–15.00) vs. 13.00 (10.00–16.00), *P* = 0.028] and higher incidence of postoperative renal impairment [49 (22.1%) vs. 72 (32.4%), *P* = 0.014]. Cardiopulmonary bypass (CPB) or non-CPB CABG patients showed similar correlations between various fibrinogen concentrations and LOS, according to subgroup analyses.

**Conclusions:**

Fibrinogen is an independent preoperative predictor of both the LOS and the postoperative renal impairment that occurs after CABG. Patients with high preoperative fibrinogen concentration had a higher incidence of postoperative renal impairment and a longer LOS, emphasizing the significance of preoperative fibrinogen management.

**Supplementary Information:**

The online version contains supplementary material available at 10.1186/s13019-023-02238-w.

## Introduction

Cardiovascular diseases (CVD) are a worldwide health care concern with escalating global trends. In 2015, the United States recorded 2,712,630 resident fatalities; the primary cause of death was CVD [1]. Similarly, in Europe, CVD is responsible for over 45% of all deaths, or more than 3,9 million deaths annually [2]. Coronary heart disease (CHD) is one of the CVD and the major cause of CVD-related deaths, accounting for 45% [1]. In both developed and developing countries, Coronary Artery Bypass Grafting (CABG) is a relatively common and successful treatment for CHD. More than 800 thousand CABG surgeries were performed all around the world in 2007 [3]. Even though CABG normally enhances survival, health, and quality of life, certain results are still unclear [4–6]. Even a significant portion of CABG patients have a difficult time recovering after surgery [5].

Despite the fact that a number of researchers have shown that short-term outcomes are vital for recovery, the majority of studies are now focused on long-term outcomes [7, 8]. Prior to this, postoperative bleeding, severe perioperative complications, and 30-day mortality were frequently employed as indications of short-term outcomes following CABG [9, 10]. Nonetheless, a newly conducted study demonstrates that hospital length of stay (LOS) is also regarded as a key indicator of surgical recovery, a surrogate for acute physical recovery, and a crucial predictor of long-term recovery [11, 12]. After CABG, patients with problems and a poor prognosis are anticipated to remain in the hospital longer. The body mass index, the use of cardiopulmonary bypass machines, the use of packed red blood cells, non-elective surgery, and the number of complications were significant predictors of longer LOS [13]. A large, multicenter investigation revealed that disparities in the diagnosis and treatment of CHD and wound infections considerably lengthen the LOS [14]. Another clinical cohort trial found that every unit increase in preoperative anxiety increased LOS by 0.381 days [15]. To the best of our knowledge, no other preoperative predictor has been established to effectively predict LOS among CABG patients.

Plasma fibrinogen, the precursor of fibrin, plays a crucial role in hemostasis by encouraging clot formation and platelet aggregation by binding to platelet glycoprotein IIb/III receptors [16]. Plasma fibrinogen level was connected with coronary heart disease, and may be a risk factor and predictor of CHD, according to a meta-analysis [17]. However, there is no research that we are aware of that looks at fibrinogen and LOS. Therefore, it is necessary to conduct a well-designed study in order to investigate the impact of fibriongen on the LOS and postoperative complications of patients who underwent CABG. To control for sample selection bias and imitate the randomization process, we apply PSM.

## Materials and methods

### Study design

The Ethical Committee of Ningbo Medical Center Lihuili Hospital approved this single-center retrospective investigation, and formal informed consent was waived. Data were obtained from consecutive patients who underwent isolated primary CABG at Ningbo Medical Center Lihuili Hospital by a single surgeon between January 2010 and June 2022. Isolated primary CABG was defined as the initial coronary artery bypass graft operation performed without or with CPB. All of the anesthesiologists who participated in the study had considerable knowledge of cardiac anesthesia. Anesthesiologists regulated blood transfusion and fluid administration in accordance with an approved fluid management standard [18].

### Study population

Adults patients (≥ 18 years) who underwent isolated primary CABG and complete medical record information were screened in the analysis. Te following exclusion criteria were used: (1) patients who underwent emergency and urgent CABG; (2) patients who underwent concurrent surgical procedures; (3) patients with liver and kidney dysfunction; (4) patients with coagulopathy; (5) patients with systemic infectious diseases; (6) patients with malignant neoplastic disease. Finally, 633 patients were enrolled in our study and these patients were categorized into high fibrinogen group and normal fibrinogen group. High fibrinogen and normal fibrinogen were defined as follows: fibrinogen < 3.5 g/L (normal fibrinogen), fibrinogen ≥ 3.5 g/L (high fibrinogen).

### Surgical procedures

(1) General combined anesthesia was administered, and the patient was placed in the supine position. (2) A standard thoracotomy was performed in the middle of the sternum, using the great saphenous vein or left internal mammary artery as the main graft vessel and the right internal mammary artery as a backup. (3) The location and number of bypass grafts were decided based on the individual conditions of patients. (4) Slowing the patient’s heart rate, reducing the contraction range, and controlling arterial blood pressure with systemic heparinization, extracorporeal circulation, and activation coagulation time > 5 min; (5) Pericardial and mediastinal drainage tubes were positioned to expose the posterior descending branch of the heart, the posterior branch of the left ventricle, or the blunt margin branch; (6) Lateral wall forceps were positioned in the ascending aorta for proximal anastomosis. (7) Protamine was administered following surgery to control the bleeding and seal the chest. After 24 h, the pericardium and mediastinal drainage tube may be withdrawn.

### Data Collection

The information was obtained from hospital medical records. We collected age, gender, smoking, family history of CHD, underlying disease (hypertension, hyperlipidemia and diabetes), unstable angina, surgery history, left ventricular ejection fraction (LVEF) and body mass index (BMI). Laboratory indicators 24 h before surgery including hemoglobin (HGB), white blood cell (WBC), neutrophil count, lymphocyte count, monocytes count, platelet (PLT) count, plateletcrit (PCT), prothrombin time (PT), activated partial thrombosis time (APTT) were recorded. We also recoded intraoperative data [CPB, surgery time, number of anastomoses, and arterial grafts] and preoperative medical therapy [low molecular weight heparin (LMWH), aspirin, clopidogrel, angiotensin-converting enzyme inhibitors (ACEI) and β-blocker]. The diagnostic criteria for unstable angina is ST segment depression or elevation on electrocardiogram and coronary angiography showing a stenosis of more than 75%. Aspirin and clopidogrel are discontinued 5 days prior to surgery; LMWH is discontinued 1 day prior to surgery; ACEI and β-blocker are taken continuously prior to surgery.

### Outcomes

The primary outcome in the present study was LOS. The secondary outcome is extraction time and postoperative complications [leg wound infections, chest infections, postoperative urinary tract infection (UTI), transient ischemic attacks (TIA), pneumonia, and renal impairment]. In addition, postoperative red blood cell (RBC) transfusion and postoperative 24 h bleeding volume. Postoperative 24 h bleeding volume was defined as the total amount of chest tube drainage within 24 h after surgery, which was recorded by an ICU-trained nurse.

### Statistical analysis

No data loss occurred for categorical variables. The loss of continuous variables was less than 5%, so mean values were substituted for missing data. Continuous variables were reported as mean ± standard deviation (for normally distributed continuous variable comparisons between groups) or median (interquartile ranges) (for non-normally distributed continuous variable comparisons between groups), and comparisons between the two groups were made using the Mann-Whitney U test or the student t test. Categorical variables are presented as total numbers, and percentages and comparisons between the two groups were made using Pearson’s chi-square test or Fisher’s exact test.

Original cohort consisted of the whole number of initial participants. In addition to the original cohort, propensity score matching (PSM) was utilized to produce well-balanced groupings, most notably the matched cohort. Using a non-parsimonious multivariable logistic regression model, the propensity score was computed with hypothermia as the dependent variable and all baseline characteristics as the independent factors. Using the greedy closest neighbor matching method with a caliper width of 0.2, patients in the high fibrinogen group were paired with patients in the normal fibrinogen group. Standardized mean differences (SMD) were computed to evaluate the efficacy of the PSM. SMD 0.1% is regarded as an acceptable compromise between the groups. Utilizing subgroup analysis, the relationship between fibrinogen concentration and LOS in CPB and non-CPB subgroups was determined.

SPSS software (version 23.0) and R software (version 4.1.1) were used for all statistical analysis. All test results were considered as statistically significant at *P* < 0.05.

## Results

### Baseline characteristics

**Fig. 1:**
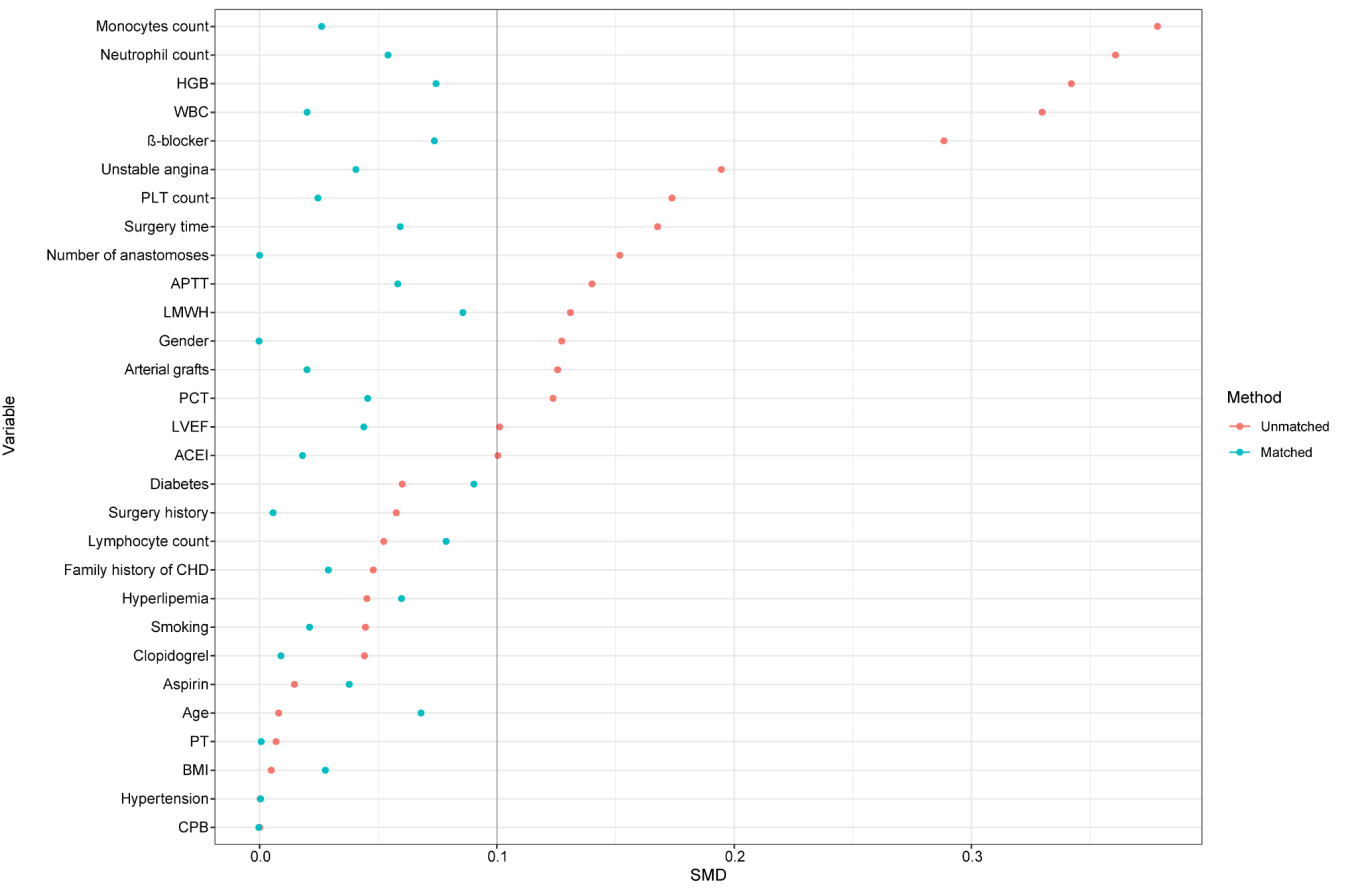
SMD between the high fibrinogen and normal fibrinogen groups in each cohort

This study enrolled a total of 633 patients, with 344 individuals in the normal fibrinogen group and 289 patients in the high fibrinogen group. The age of the study patients was 64.66 ± 8.95 years old, and 483 (76.3%) were males. After PSM, 222 patients in the normal fibrinogen group and 222 patients in the high fibrinogen group were enrolled in the PSM cohort (Table 1). Before matching, the majority of factors between the two groups were not balanced. After matching, the imbalanced covariates were balanced in the matched cohort (Table 1; Fig. 1).

**Table 1:** Baseline characteristics of subjects in the original and matched cohorts

Covariates	Original cohort			Matched cohort		
	Normal fibrinogen group	High fibrinogen group	SMD	Normal fibrinogen group	High fibrinogen group	SMD
N	344	289		222	222	
Age (years)	64.63 ± 8.84	64.70 ± 9.09	0.008	64.64 ± 9.40	65.22 ± 8.87	0.064
Men (%)	271 (78.8)	212 (73.4)	0.127	167 (75.2)	167 (75.2)	< 0.001
Smoking (%)	120 (34.9)	107 (37.0)	0.045	82 (36.9)	78 (35.1)	0.038
Family history of CHD (%)	7 ( 2.0)	8 ( 2.8)	0.048	6 ( 2.7)	5 ( 2.3)	0.029
Hypertension (%)	225 (65.4)	189 (65.4)	< 0.001	143 (64.4)	142 (64.0)	0.009
Hyperlipidemia (%)	93 (27.0)	84 (29.1)	0.045	66 (29.7)	60 (27.0)	0.06
Diabetes (%)	100 (29.1)	92 (31.8)	0.06	59 (26.6)	68 (30.6)	0.09
Unstable angina (%)	68 (19.8)	81 (28.0)	0.195	58 (26.1)	62 (27.9)	0.041
Surgery history (%)	137 (39.8)	107 (37.0)	0.058	85 (38.3)	87 (39.2)	0.018
LVEF (%)	0.60 ± 0.11	0.59 ± 0.12	0.101	0.59 ± 0.12	0.59 ± 0.12	0.045
BMI (kg /m^2^)	24.23 ± 3.07	24.25 ± 3.35	0.005	24.25 ± 3.06	24.16 ± 3.27	0.03
HGB (g/L)	132.51 ± 16.44	126.32 ± 19.57	0.342	130.32 ± 16.44	129.14 ± 16.24	0.072
WBC (×10^9^ /L)	6.10 (5.00-7.30)	6.70 (5.70-8.00)	0.33	6.50 (5.33–7.90)	6.40 (5.50–7.50)	0.02
Neutrophil count (×10^9^ /L)	3.60 (2.80–4.70)	4.30 (3.40–5.30)	0.361	3.95 (3.00–5.00)	4.00 (3.20-5.00)	0.054
Lymphocyte count (×10^9^ /L)	1.60 (1.20–2.10)	1.60 (1.20-2.00)	0.052	1.70 (1.23–2.10)	1.60 (1.20-2.00)	0.082
Monocytes count (×10^9^ /L)	0.50 (0.40–0.60)	0.60 (0.40–0.70)	0.378	0.50 (0.40–0.60)	0.50 (0.40–0.70)	0.024
PLT count (×10^9^ /L)	184.00 (158.75–224.00)	200.00 (165.00-236.00)	0.174	188.00 (163.00-235.50)	193.50 (164.00-234.00)	0.023
PCT (%)	0.17 (0.15–0.20)	0.18 (0.15–0.21)	0.124	0.18 (0.15–0.20)	0.18 (0.15–0.21)	0.044
PT (sec)	11.56 ± 0.93	11.55 ± 1.35	0.007	11.53 ± 0.91	11.53 ± 1.29	0.007
APTT (sec)	32.23 ± 4.12	33.08 ± 7.49	0.14	32.41 ± 4.42	32.68 ± 5.13	0.056
CPB (%)	50 (14.5)	42 (14.5)	< 0.001	29 (13.3)	29 (13.3)	< 0.001
Surgery time (h)	290.00 (255.00-330.00)	299.00 (265.00-340.00)	0.168	295.00 (257.25-332.75)	298.50 (261.25-340.75)	0.056
Number of anastomoses	3.01 ± 0.81	3.13 ± 0.84	0.152	3.11 ± 0.75	3.10 ± 0.90	0.005
Arterial grafts	263 (76.5)	205 (70.9)	0.126	160 (72.1)	158 (71.2)	0.02
LMWH(%)	324 (94.2)	280 (96.9)	0.131	215 (96.8)	214 (96.4)	0.025
Aspirin (%)	213 (61.9)	181 (62.6)	0.015	137 (61.7)	137 (61.7)	< 0.001
Clopidogrel (%)	165 (48.0)	145 (50.2)	0.044	114 (51.4)	110 (49.5)	0.036
ACEI (%)	153 (44.5)	143 (49.5)	0.1	102 (45.9)	100 (45.0)	0.018
β-blocker (%)	214 (62.2)	218 (75.4)	0.288	164 (73.9)	157 (70.7)	0.071

CHD: coronary heart disease, LVEF: left ventricular ejection fraction, BMI: body mass index, HGB: hemoglobin, WBC: white blood cell, PLT: platelet, PCT: plateletcrit, PT: prothrombin time, APTT: activated partial thromboplastin time, CPB: cardiopulmonary bypass, LMWH: low molecular weight heparin, ACEI: angiotensin-converting enzyme inhibitors

### Primary outcomes with different cohorts

In total, the median LOS of the study patients was 12.00 (9.00–15.00). The Mann-Whitney U test showed that the high fibrinogen group had a longer LOS [11.00 (9.00–14.00) vs. 13.00 (10.00–16.00), *P* < 0.001] than the normal fibrinogen group in the original cohort. In the matched cohort, the high fibrinogen group had a longer LOS than the normal fibrinogen group [12.00 (9.00–15.00) vs. 13.00 (10.00–16.00), *P* = 0.028] (Table 2).

**Table 2:** Short-term Outcomes of subjects in the original and matched cohorts

Covariates	Original cohort			Matched cohort		
	Normal fibrinogen group	High fibrinogen group	*P*	Normal fibrinogen group	High fibrinogen group	*P*
N	344	289		222	222	
LOS	11.00 (9.00–14.00)	13.00 (10.00–16.00)	< 0.001	12.00 (9.00–15.00)	13.00 (10.00–16.00)	0.028
Extraction time	1.00 (1.00–2.00)	1.00 (1.00–2.00)	0.152	1.00 (1.00–2.00)	1.00 (1.00–2.00)	0.442
leg wound infections	6 (1.7%)	5 (1.7%)	1.000	3 (1.4%)	2 (0.9%)	1.000
Chest infections	1 (0.3%)	1 (0.3%)	1.000	1 (0.5%)	1 (0.5%)	1.000
Postoperative UTI	1 (0.3%)	0 (0%)	1.000	1 (0.5%)	0 (0%)	1.000
TIA	0 (0%)	2 (0.7%)	1.000	0 (0%)	1 (0.5%)	1.000
Pneumonia	10 (2.9%)	5 (1.7%)	0.437	6 (2.7%)	3 (1.4%)	0.503
Renal impairment	70 (20.3%)	86 (29.8%)	0.006	42 (18.9%)	72 (32.4%)	0.014
Postoperative RBC transfusion	160 (46.5%)	148 (51.2%)	0.272	106 (47.7%)	118 (53.2%)	0.296
Postoperative 24 h bleeding volume	597.50 (473.75–820.00)	555.00 (440.00-720.00)	0.002	577.50 (461.25–807.50)	567.50 (466.25–750.00)	0.369

LOS: length of stay; UTI: urinary tract infection; TIA: transient ischemic attacks; RBC: red blood cell

### Secondary outcomes with different cohorts

There was no significant difference in postoperative extraction time, leg wound infections, chest infections, postoperative UTI, TIA, pneumonia, and postoperative RBC transfusion between the two groups (*P* > 0.05). The Mann-Whitney U test showed that the high fibrinogen group had a less postoperative 24 h bleeding volume [597.50 (473.75–820.00) vs. 555.00 (440.00-720.00), *P* = 0.002] than the normal fibrinogen group in the original cohort. In the matched cohort, there was no significant difference in postoperative 24 h bleeding volume between the two groups (577.50 (461.25–807.50) vs. 567.50 (466.25–750.00), *P* = 0.369). The high fibrinogen group had a higher Incidence of postoperative renal impairment than the normal fibrinogen group in the original cohort [70 (20.3%) vs. 86 (29.8%), *P* = 0.006]. In the matched cohort, the high fibrinogen group had a higher incidence of postoperative renal impairment [42 (18.9%) vs. 72 (32.4%), *P* = 0.014] (Table 2).

### Subgroup analyses

Subgroup analyses for the correlation between different fibrinogen concentration and LOS were presented in Table 3. The participants were divided into subgroups according to the patients who used CPB and those who did not. The results showed that the association between different fibrinogen concentration and LOS stably existed in the subgroups (Table 3, *P* < 0.05). the association between different fibrinogen concentration and incidence of postoperative renal impairment also stably existed in the subgroups (Table 4, *P* < 0.05).

**Table 3:** Comparison of LOS in different fibrinogen concentration levels subgroups in CPB and non-CPB groups

Group	N	LOS	*P*
**CPB**			0.007
**Normal fibrinogen group**	29	13.00 (10.00–18.00)	
**High fibrinogen group**	40	20.00 (14.00–26.00)	
**Non-CPB**			0.019
**Normal fibrinogen group**	193	12.00 (9.00–15.00)	
**High fibrinogen group**	182	15.00 (10.00–19.00)	

**Table 4:** Comparison of renal impairment in different fibrinogen concentration levels subgroups in CPB and non-CPB groups

Group	N	Renal impairment	*P*
**CPB**			0.043
**Normal fibrinogen group**	29	5 (17.2%)	
**High fibrinogen group**	40	16 (40.0%)	
**Non-CPB**			0.009
**Normal fibrinogen group**	193	37 (19.2%)	
**High fibrinogen group**	182	56 (30.8%)	

## Discussion

In present study, we demonstrated the association of preoperative high fibrinogen concentration and the longer LOS. We have showed in this study that preoperative high fibrinogen concentration is prevalent among patients who underwent CABG, with roughly a half of our sample. Despite applying the PSM, patients in the high-fibrinogen group still showed longer LOS and a higher incidence of postoperative renal impairment than those in the normal fibrinogen group. Furthermore, the results were consistent across subgroups based on CPB and non-CPB subgroups.

Fibrinogen’s role as a predictor of LOS has not before been described. Previous research have mostly focused on the correlation between fibrinogen and the occurrence and development of CHD [19–21]. Fibrinogen is one of the most accurate predictors of coronary heart disease, which is related to the occurrence of CHD [19]. Reducing fibrinogen level may be a viable strategy for lowering CHD risk [20]. In addition, fibrinogen, a marker of inflammation and coagulation, may contribute to the increased all-cause mortality associated with CHD [21]. Several molecular pathways are markedly and persistently activated following CABG, showing elevated inflammatory state, hemostasis activation, and oxidative stress [22]. Not only did fibrinogen have this influence on postoperative recovery, but the higher the level of circulating fibrinogen, the worse the decline in pulmonary function following CABG [23]. Inflammation is necessary for eliminating infection and debris following surgery, but if it lasts too long, it can cause tissue damage [24]. This can result in an increase in scarring or chronic wounds, extending LOS. This can result in worse scarring or chronic wounds, which, like wound infection, can lengthen LOS [14]. However, there was no statistically significant difference in postoperative infections (leg wound infection, and chest infection) between patients with high fibrinogen and those with normal fibrinogen, which may be related to the milder condition of the patients included in our study.

We found that preoperative hyperfibrinogen was a major indicator of increased postoperative renal impairment, which is consistent with other findings of similar nature [25, 26]. Renal impairment is a common and serious complication after cardiac surgery, which not only significantly extends the LOS but also significantly increases the risk of death [27, 28]. The pathogenesis of renal impairment is complex and highly specific in the coagulation and fibrinolytic systems. Studies have shown that fibrinogen is elevated in chronic kidney disease [29]. A clinical study has reported that fibrinogen deposition in renal tissue after ischemic reperfusion injury and urine fibrinogen is an early indication of renal impairment in patients undergoing abdominal aortic aneurysm repair [25]. In addition, celik et al. found that elevated serum fibrinogen levels at baseline were associated with the development of contrast media-induced renal impairment [30].

Despite our best efforts, numerous limitations persist. Our initial study was conducted in a single Chinese patient facility, which may not be representative of the entire population. Due to the retrospective study design, selection bias could not be eliminated. The PSM methodology was used to confirm the validity of our findings. Thirdly, some other variables, such as C-reactive protein, which played significant roles in evaluating the severity of inflammation, were unavailable due to a massive number of missing values.

This study demonstrated a correlation between preoperative fibrinogen concentration and LOS and postoperative complications. Fibrinogen may be used as a predictor of LOS and postoperative renal impairment. In order to provide a more accurate preoperative prognosis of CABG outcomes and to obtain greater economic benefits, we strongly advise taking fibrinogen levels into account during preoperative examination.

## Electronic supplementary material

Below is the link to the electronic supplementary material.

Additional File: fib Ethical approval

## Data Availability

The datasets used and analysed during the current study are available from the corresponding author on reasonable request.

## Glossary of abbreviations

ACEI: angiotensin-converting enzyme inhibitors
BMI: body mass index
CABG: coronary artery bypass grafting
CVD: cardiovascular disease
CHD: Coronary heart disease
CPB: cardiopulmonary bypass
HGB: hemoglobin
LOS: length of stay
LMWH: low molecular weight heparin
LVEF: left ventricular ejection fraction
PLT: platelet
PSM: propensity score matching
RBC: red blood cell
SMD: Standardized mean differences
TIA: transient ischemic attacks
UTI: urinary tract infection
WBC: white blood cell
