# Supplementary material for: Fibrinogen is an independent preoperative predictor of hospital length of stay among patients undergoing coronary artery bypass grafting
Source: J Cardiothorac Surg. 2023 Apr 7;18:112. doi: 10.1186/s13019-023-02238-w (PMC10082530; doi:10.1186/s13019-023-02238-w)
Supplement: Supplementary file 1 — Additional File: fib Ethical approval [file 13019_2023_2238_MOESM1_ESM.pdf]

伦理审查批件
Approval Letter of the Ethics Committee

批件编号 Approval Number	KY2022PJ104	项目受理号 Acceptance number	KY2022SL104-01
项目名称 Project	围术期因素对冠状动脉搭桥患者术后出血的影响		
研究类型 Project Category	□药物临床试验 □医疗器械临床试验 □体外诊断试剂临床试验 □科研项目 □新技术新项目 ■其他 研究者发起的研究		
申办单位 Sponsor	宁波市医疗中心李惠利医院		
组长单位 Group Leader	无		
主要研究者 Principal Investigator	黄春晟	申请专业 Department	兴宁-麻醉
审查类别 Category of Review	■初始审查 □复审		
审查方式 Type of Review	□会议审查 □紧急会议审查 ■快速审查		
审查日期 Date of Review	2022年4月17日	审查会议地点 Location of Review	无
审查文件 Reviewed Items	见附件		
审查意见 Evaluation	1、基本符合 GCP 原则，同意进行临床研究。<br>2、持续审查频度为研究批准之日起： □3个月 ■6个月 □1年<br>3、医学伦理委员会有权根据实际进展情况改变持续审查频度。		

主任/副主任委员签名：[signature] 日期：2022.4.25

医学伦理委员会（盖章）

注意【请仔细阅读】：
1、研究者应遵循伦理委员会同意的方案执行，实施过程应符合赫尔辛基宣言和 GCP 的基本原则。
2、在研究实施过程中，对研究方案和知情同意等相关文件所做的任何修改，均需得到伦理委员会审查同意后方可实施。
3、发生严重不良事件及可能影响风险受益比的任何事件和新信息需及时报告伦理委员会。
4、接受伦理委员会持续审查的项目，请在到期前 1 个月（无论研究开始与否）提出持续审查的申请。
5、如有违背/偏离方案或暂停/提前终止的研究项目，应及时以书面文件报告伦理委员会。
6、临床研究结束后，须及时向伦理委员会提交结题报告。
7、本批件有效期 1 年（自批准之日起），如研究逾期未实施即自行废止。
8、本批件一式二份，分别由主要研究者和本伦理委员会保存。

附件：

项目名称<br>Project	围术期因素对冠状动脉搭桥患者术后出血的影响
审查文件<br>Reviewed Items	(1) 伦理审查申请书（申请免除知情同意）；<br>(2) 主要研究者履历；<br>(3) 研究方案（版本号：V1.0 ，日期：2022 月 03 月 20 日）；<br>(4) 研究资料登记表（版本号：V1.0 ，日期：2022 月 03 月 20 日）。
医学伦理委员会（盖章）	

地址：宁波市兴宁路 57 号 邮编：315040 联系人：章培 0574-87018834/0574-55835593 邮箱：lihuiliethics@163.com
